# Visible-Light-Curable Catechol-Reinforced Gelatin Bioglue for Rapid Hemostasis and Engineered Tissue Assembly

**DOI:** 10.34133/bmr.0360

**Published:** 2026-05-07

**Authors:** Ashfaq Ahmad, Jian Shin, Jaylord M. Pioquinto, Se Eun Kim, Yong Sook Kim, Jessie S. Jeon, Yeong-Jin Choi, Hee-Gyeong Yi

**Affiliations:** ^1^ Department of Convergence Biosystems Engineering, College of Agriculture and Life Sciences, Chonnam National University, Gwangju, Republic of Korea.; ^2^ Interdisciplinary Program in IT-Bio Convergence System, Chonnam National University, Gwangju, Republic of Korea.; ^3^ Department of Veterinary Surgery, College of Veterinary Medicine, Chonnam National University, Gwangju, Republic of Korea.; ^4^ Biomedical Research Institute, Chonnam National University Hospital, Gwangju, Republic of Korea.; ^5^ Medical Research Center, Chonnam National University Medical School, Hwasun, Republic of Korea.; ^6^ Department of Mechanical Engineering, Korea Advanced Institute of Science and Technology (KAIST), Daejeon, Republic of Korea.; ^7^ Advanced Bio and Healthcare Materials Research Division, Korea Institute of Materials Science (KIMS), Changwon, Republic of Korea.; ^8^ Advanced Materials Engineering, Korea National University of Science and Technology (UST), Daejeon, Republic of Korea.; ^9^ Institute for Biomedical Science, Chonnam National University Hospital Hwasun, Hwasun, Republic of Korea.

## Abstract

Hydrogel-based tissue adhesives hold promise for diverse biomedical applications, yet achieving strong, durable adhesion to wet, dynamic biological surfaces remains challenging. The catechol-containing amino acid 3,4-dihydroxy-l-phenylalanine (DOPA) is a crucial functional motif in mussel-inspired adhesives and has been extensively investigated for incorporation into various biomaterials. However, conventional approaches of DOPA incorporation and subsequent crosslinking have shown limited potential for clinical translation. We developed a bioinspired strategy involving pH-controlled enzymatic conversion of tyrosine residues in porcine gelatin into DOPA. The resulting catechol-functionalized gelatin was blended with native tyrosine-rich gelatin to form a hybrid adhesive hydrogel, termed DOPA–tyrosine gelatin (DTG) bioglue. DTG was designed to achieve (a) rapid visible-light-driven ruthenium-based crosslinking for structural integrity and (b) enhanced interfacial adhesion via DOPA-mediated interactions with tissue surface. Its shear-thinning and fast-curing properties enable its use as a bioink for both extrusion-based and digital-light-processing 3-dimensional bioprinting. We demonstrated the DTG-assisted tissue–tissue integration for large-area construct assembly, addressing current volumetric fabrication limitations in microextrusion bioprinting. Moreover, it demonstrated superior hemostatic performance in rat liver and vascular injury models. Collectively, DTG bioglue is a promising candidate for next-generation adhesive biomaterial for regenerative medicine and surgical applications.

## Introduction

Hydrogel-based bioadhesives are natural or synthetic polymers that serve as “glue” to bond with living tissues [1]. They have gained substantial research interest due to their biocompatibility, degradability, tunable mechanical properties, and biomimetic characteristics, making them ideal for diverse applications in clinical and engineering fields, including wound closure, surgical sealants [2,3], regenerative medicine [4,5], drug delivery [6], trauma care, and implantable wearable devices [7]. Hydrogel-based tissue adhesives offer advantages such as simplicity, rapid application, reduced patient trauma, and minimized complications in surgical and regenerative settings [8]. Despite their promising properties, hydrogel-based bioadhesives still face a couple of challenges, particularly in achieving strong mechanical integrity and reliable adhesion to wet biological tissues.

Achieving strong adhesion to biological surfaces, particularly those involving blood or other fluids, is a critical requirement for hydrogel-based tissue sealants. However, water can interfere with adhesion by forming a hydration layer on tissue surfaces and disrupting essential inter- and intramolecular interactions, often resulting in interfacial or bulk failure and reduced bonding strength [9]. For example, fibrin sealants, which are U.S. Food and Drug Administration-approved in-situ-forming adhesives widely used for hemostasis and tissue repair, demonstrate limited mechanical performance and suboptimal adhesion under such conditions [10]. Moreover, concerns regarding viral transmission persist despite the implementation of rigorous viral screening and inactivation processes. In contrast, synthetic adhesives like cyanoacrylates [11] provide rapid bonding and high tensile strength, yet their clinical translation is limited by several drawbacks, including cytotoxicity, rigidity, opacity, poor flexibility, and heat generation during polymerization, which can damage surrounding tissues [12].

To overcome the limitations associated with weak wet adhesion and insufficient cohesive strength, substantial research efforts, largely inspired by marine organisms, have focused on mitigating the adverse effects of interfacial water and reinforcing the mechanical integrity of bioadhesives [13]. Among marine species, mussels have served as a model for developing wet-resistant adhesives, owing to their ability to firmly attach to various submerged surfaces [14]. This remarkable adhesion is primarily attributed to the presence of 3,4-dihydroxy-l-phenylalanine (DOPA) residues within mussel foot proteins [15,16]. DOPA plays a multifaceted role in underwater adhesion by facilitating hydrogen bonding, π–cation and π–π interactions, and metal coordination (e.g., with Fe^3+^) and promoting localized hydrophobicity to displace interfacial water layers [17]. These interactions collectively enable strong and reversible adhesion under wet and dynamic biological conditions. Inspired by these natural mechanisms, various DOPA-functionalized polymers to mimic mussel adhesive properties have been developed [18–21]. DOPA moieties have been chemically grafted onto a wide range of natural and synthetic polymers, including alginate, hyaluronic acid [22], chitosan [23], heparin [24], and poly(ethylene glycol) [25], thereby imparting them with enhanced adhesion capabilities suitable for biomedical applications such as tissue sealants, wound-closure systems, and implantable hydrogels. However, the incorporation of DOPA into polymer backbones poses considerable challenges. The synthesis typically involves costly and labor-intensive multistep chemical modification and purification processes. Moreover, during synthesis and storage, DOPA is prone to spontaneous oxidation to DOPA-quinone, which can undergo further polymerization into melanin-like species [26]. This oxidative conversion not only alters the chemical structure but also reduces the availability of free catechol groups essential for adhesion, ultimately lowering the bioadhesive performance of the resulting material. In addition to these synthetic challenges, the commonly used cross-linking strategies, such as ultraviolet (UV) light activation [19], pH-triggered gelation [27], and cation-based [28,29] or chemical cross-linkers, introduce further limitations. These methods often involve the generation of reactive species or require harsh conditions that may lead to cytotoxic effects or unintended tissue damage, thereby reducing their clinical translatability. Consequently, bioinspired strategies for DOPA functionalization that employ biocompatible and scalable cross-linking methods remain a critical priority in the design of next-generation, DOPA-based bioadhesives suitable for clinical applications.

**Fig. 1.**
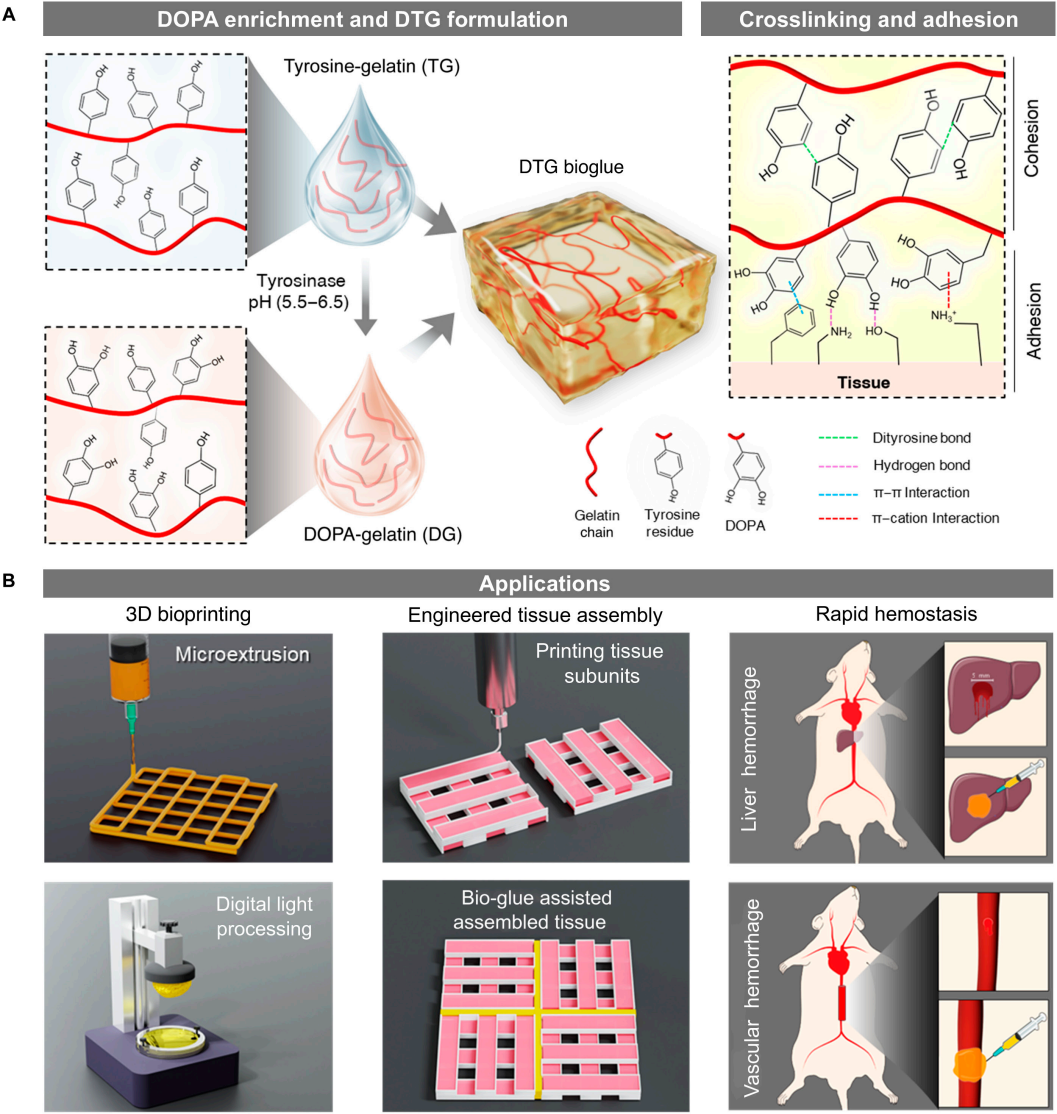
Overview of the study. (A) Schematic illustration of 3,4-dihydroxy-l-phenylalanine (DOPA) functionalization of gelatin, formulation of DOPA–tyrosine gelatin (DTG) bioglue, and its cross-linking and adhesion mechanisms. (B) Demonstrated applications, including 3-dimensional (3D) bioprinting, engineered tissue assembly, and rapid hemostasis in liver and vascular hemorrhage models.

Inspired by the natural biosynthesis of DOPA, this study introduces a straightforward strategy involving the pH-controlled enzymatic hydroxylation of tyrosine residues in gelatin using tyrosinase. Porcine-derived gelatin was selected as the base material due to its high tyrosine content, approximately 2.6% of tyrosine as compared to 1% of that reported in bovine-derived gelatin [30]. The resulting DOPA-enriched gelatin was subsequently blended with unmodified gelatin containing native tyrosine residues to form a DOPA–tyrosine gelatin (DTG) bioglue. This formulation was optimized to satisfy 2 most critical aspects of an ideal bioglue: (a) rapid photocrosslinking via visible-light-activated ruthenium (Ru)-based photoinitiation, providing structural integrity; and (b) interfacial adhesion enhancement through DOPA-mediated interactions. The catechol groups in DOPA facilitate strong wet adhesion through multiple bonding mechanisms, including hydrogen bonding, π–π interactions, and metal coordination, thereby improving the adhesive performance under physiological conditions (Fig. 1A).

The DTG bioglue demonstrated significantly enhanced adhesive properties compared to both commercial fibrin glue and unmodified gelatin. In addition, its shear-thinning behavior and rapid visible-light-induced cross-linking make it particularly well suited for microextrusion and digital light processing (DLP)-based bioprinting techniques, respectively. We utilized the DTG bioglue to facilitate the assembly of modular engineered tissues into larger constructs, a persistent challenge in microextrusion bioprinting due to extended printing times and structural instability. Furthermore, the hemostatic potential of the DTG bioglue was evaluated in vivo using rat models of vascular and hepatic hemorrhage, where it again outperformed fibrin glue in achieving rapid and effective bleeding control (Fig. 1B). Taken together, these findings establish DTG bioglue as a promising candidate for next-generation adhesive biomaterials with broad potential in regenerative medicine, bioprinting-based tissue fabrication, and surgical hemostasis.

## Materials and Methods

### Synthesis and formulation of the DTG bioglue

Gelatin from porcine skin (300 g of Bloom strength; Sigma-Aldrich, USA) was dissolved at a concentration of 10% (w/v) in 1× phosphate-buffered saline (PBS; Biosesang, Korea) by incubating at 37 °C for 1 h under gentle agitation. The pH of the solution was adjusted to 5.5 to 6.5 using 1 M HCl (Biosesang). Mushroom tyrosinase (Sigma-Aldrich) was then added to the gelatin solution and incubated at 37 °C for a predetermined duration. To inactivate the enzyme, the solution was heated at 60 °C for 1 h. The resulting tyrosinase-treated gelatin solution was labeled as DOPA-gelatin (DG) and stored at −80 °C until use. Prior to formulation of the DTG bioglue, DG was thawed at 37 °C for 30 min and mixed with unmodified gelatin (TG) at a specific ratio. The blended solution was maintained at 37 °C until further use. Photoinitiator stock solutions of Ru (Sigma-Aldrich) and sodium persulfate (SPS; Sigma-Aldrich) were prepared at concentrations of 50 and 500 mM, respectively. These were added to the bioglue formulation at final concentrations of 0.5 mM (Ru) and 5 mM (SPS). The mixture was then exposed to white light to initiate cross-linking and form the hydrogel.

### Quantification of DOPA contents

DOPA content was quantified using Arnow’s colorimetric method. A nitrite–molybdate reagent was freshly prepared by dissolving 1 g of sodium nitrite (Sigma-Aldrich) and 1 g of sodium molybdate dihydrate (Sigma-Aldrich) in 10 ml of ultrapure water. Prior to the assay, each sample was diluted 10-fold using distilled water. To each 1 ml of the diluted sample, 1 ml of 0.5 M HCl was added, followed by the addition of 1 ml of the freshly prepared sodium nitrite–molybdate solution. The mixture was thoroughly mixed, and then 1 ml of 1 M NaOH was added. The final volume of the reaction mixture was adjusted to 5 ml using distilled water. The reaction was allowed to proceed at room temperature for 30 min in the dark. A standard curve was generated using known concentrations of 3,4-dihydroxybenzoic acid (Sigma-Aldrich). Absorbance of all samples and standards was measured at 500 nm using a plate reader. UV–visible spectra of DOPA-modified gelatin or enzymatically digested hydrogel after cross-linking were recorded in quartz cuvette.

### Ex vivo adhesion and mechanical strength testing of DTG

Fresh porcine aortic tissues, presoaked in PBS, were trimmed to desired size for each test. For the lap-shear test, tissue samples were securely affixed onto standard glass microscope slides using cyanoacrylate glue. An amount of DTG bioglue, prepared in various formulations, was applied between the overlapping areas of tissue (20 mm × 20 mm). For the peel-strength test, bioglue was applied over a larger overlapping region and tissues were cut into 30-mm-wide pieces. In the tensile-strength test, equal-sized aortic samples were bonded end-to-end using the bioglue. The jointed area was irradiated with an ordinary light-emitting diode (LED) flashlight for 30 s to induce cross-linking. As a control, fibrin glue was prepared in-house by mixing fibrinogen and thrombin (Sigma-Aldrich) immediately prior to application. Samples were mounted onto a universal testing machine and pulled in tension at a crosshead speed of 10 mm/min until failure. Adhesive strength and shear strength were calculated by dividing the maximum force sustained before failure by the contact area of the bioglue and tissue. Peeling strength was determined by dividing the plateau force required to peel the tissue by the tissue width.

To evaluate the sealing performance of the bioglue under pressure, a custom burst-pressure testing apparatus was fabricated. The setup consisted of a 3-dimensional (3D) printed open-top air chamber, onto which a square piece of porcine aortic tissue was securely mounted using cyanoacrylate glue (Fig. S1A and B). The air chamber was connected to a regulated air supply and a pressure gauge. A linear incision was made on the center of the mounted tissue surface and immediately sealed using the bioglue. After curing the bioglue with visible light for 30 s, air was purged into the chamber until leakage or failure of the adhesive seal was observed, defined as the burst pressure.

After initial mechanical screening to identify the optimal DTG formulation, additional qualitative tests were performed. The optimized DTG bioglue was applied to the wet surface of porcine aortic tissue, which was then subjected to physical deformation including stretching, twisting, bending, and exposure to water flushing to evaluate adhesive robustness under dynamic conditions. Porcine skin samples were bonded using either DTG bioglue or fibrin glue. The adhered constructs were immersed in water and PBS at 37 °C for 72 h on a shaker to assess adhesion stability under wet incubation.

### Ultrastructure analysis

To observe the microstructure of the hydrogels and the tissue–adhesive interface, TG and DTG hydrogel samples were first prepared and thoroughly washed 3 times with distilled water. The samples were then frozen at −80 °C. To examine the interface between the DTG bioglue and biological tissue, the bioglue was applied onto fresh porcine aortic tissue, followed by freezing at −80 °C overnight. All frozen samples were lyophilized under vacuum conditions for 72 h and trimmed to expose the internal structure and interface regions. Samples were sputter coated with a thin layer of gold, and scanning electron microscopy (SEM) imaging was conducted at an acceleration voltage of 10 kV.

### Rheological assessment

The rheological properties of the hydrogels were characterized using a rotational rheometer (TA Instruments, USA) equipped with customized irradiation setup (Fig. S2A). For each measurement, a hydrogel was loaded onto the lower plate, and the upper plate was lowered to reach the desired gap. A time-sweep test was performed to monitor the evolution of the storage (*G*′) and loss (*G*″) moduli during visible-light-induced photocrosslinking. After 100 s, light irradiation was initiated manually and maintained until 600 s. This test was used to assess the phase transition behavior of TG and DTG hydrogels under illumination. To assess shear-thinning behavior, a flow-sweep test was performed by measuring viscosity as a function of increasing shear rate from 0.1 to 1,000 s^−1^.

### Light-source characterization

High-resolution emission spectra of the handheld LED flashlight and the flashlight of the Galaxy S22 Ultra (Samsung) were recorded using a fiber-coupled StellarNet Blue-Wave spectrometer system in scope mode (StellarNet Inc.). The spectral irradiance profile of the LED flashlight was measured at a fixed distance of 5 cm. Total irradiance (integrated irradiance) of more than 400 to 600 nm was quantified using a calibrated photodiode sensor, and distance-dependent integrated irradiance profiles were also obtained. For in vitro and in vivo experiments, the handheld LED flashlight was used to irradiate samples at ~5 cm, corresponding to an integrated irradiance of approximately 5 mW/cm^2^ (Fig. S2B and C).

### Printability assessment

To evaluate the extrusion-based printability of the bioink, printing was performed using a pneumatic-driven multihead bioprinter (T&R Biofab, Korea). The DTG bioink was loaded into a 3-ml sterile disposable syringe. The bioink was extruded into a predefined pattern under varying pressure conditions to determine the optimal printability. Printing accuracy was assessed by comparing the dimensions of the printed lattice structures with the corresponding theoretical design. A ratio between 0.8 and 1.2 was considered to fall within acceptable range, indicating acceptable shape fidelity.

DLP-based bioprinting was conducted using a custom-designed microprojection stereolithography system (LITHO MICRO, Plancklab Inc., Korea), engineered for high-precision, bottom-up photopolymerization at the microscale. The optical engine consisted of a 405-nm near-UV LED and a 16.5-mm (0.65-in.)digital micromirror device module with full high-definition resolution (1,920 × 1,080), projecting a pixel pitch of 35 μm onto the build plane. The system supports a maximum build volume of 67.2 mm × 37.8 mm × 50 mm, and the light intensity at the bioink interface was set to 30 mW/cm^2^ during printing. The bioink vat consisted of a polystyrene Petri dish coated with polydimethylsiloxane, providing a low-adhesion surface that facilitates smooth layer separation. A borosilicate glass substrate was used as the build platform due to its excellent optical transparency, thermal resistance, and chemical stability. Prior to printing, the build platform was treated with oxygen plasma to enhance adhesion between the printed construct and the substrate.

Approximately 2 ml of bioink was loaded into the polydimethylsiloxane-coated vat, and the ambient temperature during printing was maintained at 37 °C to prevent physical entanglement of gelatin chains. 3D models were obtained from the National Institutes of Health (NIH) 3D Models Library and processed using slicing software to generate layer-by-layer projection instructions. Key printing parameters, including layer thickness (typically 50 μm), exposure time (15 s per layer), lifting distance, and lifting speed, were configured using custom control software and could be adjusted on a per-layer basis for adaptive optimization. After printing, the fabricated constructs were gently rinsed with PBS to remove any uncross-linked bioink residues.

### Hemocompatibility test

Fresh human whole blood from a consented donor was collected in 10-ml sodium heparin (NaHep) Vacutainer tubes (Custom, Whole Blood, Goma Biotech). The blood was centrifuged at 1,500 rpm for 10 min to pellet the red blood cells (RBCs). The supernatant was removed, and the RBC pellet was carefully washed 3 times with PBS. Hydrogel samples were preconditioned by immersing them in PBS to obtain hydrogel-conditioned media. Pelleted RBCs were mixed with 1 ml of the hydrogel-conditioned PBS solution. The mixture was centrifuged at 12,000 rpm for 15 min to separate intact cells from released hemoglobin in the supernatant. The absorbance of the supernatant was measured at 540 nm.

### Cell culture

Human umbilical vein endothelial cells (HUVECs; PromoCell, Germany) were cultured in VascuLife endothelial cell growth medium (Lifeline Cell Technology, USA) according to the supplier’s protocol. Cultures were maintained in a humidified incubator at 37 °C with 5% CO_2_, and the growth medium was replenished every other day. HepG2 cells (American Type Culture Collection, USA) were cultured in minimum essential medium (American Type Culture Collection) supplemented with 10% fetal bovine serum (Gibco, USA) and 1% penicillin–streptomycin (Gibco). Prior to use, the complete medium was sterilized by filtration through a 0.22-μm membrane filter (Millipore, USA). Culture dishes were coated with collagen (50 μg/ml) for 30 min before seeding to promote attachment.

### Cytocompatibility test

The cytocompatibility of DTG bioglue was evaluated through in vitro culture HUVECs under both direct and indirect contact conditions. For the direct-contact test, disc-shaped hydrogel samples were prepared by casting the hydrogel into a 3D-printed mold. The resulting hydrogel discs were carefully placed onto HUVECs cultured in a 24-well plate (SPL, Korea). For the indirect contact assay, the hydrogel discs were incubated in complete endothelial cell culture medium for 24 h to allow the leaching of any soluble components. The resulting hydrogel-conditioned medium was then collected and applied to HUVECs cultured in a 24-well plate.

For live/dead staining, an assay solution containing 10 μM 5-carboxyfluorescein diacetate (Sigma-Aldrich) and propidium iodide (2 μg/ml; Sigma-Aldrich) was prepared. After removing the hydrogel and medium, the staining solution was added to each well and incubated for 30 min at 37 °C. Cells were then washed twice with sterile Dulbecco’s PBS (Invitrogen, USA). Fluorescence images were captured using a fluorescence microscope (Zeiss, Germany). The number of live (green) and dead (red) cells was quantified from randomly selected images using ImageJ software (NIH, USA), and cell viability was calculated as the percentage of live cells over the total number of cells. Cell proliferation was further assessed using the Cell Counting Kit-8 (CCK-8; Dojindo, Japan), following the manufacturer’s protocol.

Endothelial monolayers were fixed on day 14. After blocking with 5% bovine serum albumin, the samples were incubated overnight at 4 °C with primary antibody (anti-CD31; 1: 50 dilution; Invitrogen, MA5-13188). Following washing, the samples were incubated with Alexa-Fluor-488-conjugated secondary antibody (1:200 dilution; Invitrogen), counterstained with 4′,6-diamidino-2-phenylindole (1:500 dilution; Invitrogen, D1306), and imaged using a fluorescence microscope.

### Bioink preparation and bioprinting of liver constructs

Collagen sponge (TDM, Korea) was digested in 10 mM HCl for 48 h at 4 °C to obtain a solubilized collagen solution. After complete digestion, the solution was neutralized using 1 M NaOH, followed by the addition of 10× PBS (Biosesang) and distilled water to achieve the desired concentration. The neutralized collagen solution was stored at 4 °C for up to 1 month. To prepare the liver bioink, HepG2 cells were harvested and pelleted. The cell pellet was resuspended in the collagen solution by gently pipetting up and down to achieve a final cell density of 5 × 10^6^ cells/ml. The resulting cell-laden bioink was loaded into a sterile 3-ml disposable syringe fitted with a 27-gauge needle and maintained at 15 °C during the bioprinting process. For structural support, polycaprolactone (molecular weight, 50,000; Polysciences, USA) was melted at 90 °C for 10 min and extruded at a pressure of 350 kPa to fabricate the supporting framework. The liver bioink was alternately deposited between the polycaprolactone (PCL) struts to form a composite structure.

### Cell viability and proliferation tests

Tissue constructs were gently washed with PBS on day 1 and day 4 after fabrication. Constructs were then incubated with 5-carboxyfluorescein diacetate and propidium iodide staining solution as described earlier. The stained constructs were observed under a fluorescence microscope, and images were captured from multiple randomly selected regions of interest and analyzed using ImageJ to calculate the cell viability. Cell proliferation was assessed using the CCK-8 kit, following the manufacturer’s instructions. Absorbance was measured at 450 nm using a microplate reader to determine the relative cell metabolic activity.

### Liver-function tests

To evaluate liver-specific functionality, culture media were collected from single-session bioprinted and assembled liver tissue constructs on days 3, 5, and 7 postfabrication. Secreted albumin levels were measured using a human-specific enzyme-linked immunosorbent assay kit (Sigma-Aldrich), following the manufacturer’s protocol. Urea concentration was determined using a colorimetric urea assay kit (Abcam, UK), also according to the supplier’s instructions. All absorbance measurements were performed using a microplate reader (SpectraMax, Molecular Devices, USA).

### Hydrogel characterization

To evaluate the swelling properties of the hydrogel, the initial weight of freeze-dried, cross-linked hydrogel samples was recorded. The samples were then immersed in PBS and allowed to swell until equilibrium was reached. At specified time points, the swollen hydrogels were gently blotted with filter paper to remove excess water and weighed to calculate the swelling percentage.

In vitro degradation of the hydrogel was assessed by submerging preweighed, freeze-dried hydrogel samples in PBS or collagenase solution. At predetermined time points, samples were collected, washed 3 times with deionized water, freeze-dried, and weighed to determine the remaining mass.

For in vivo degradation studies, mice were anesthetized, and a 1-cm dorsal incision was made to create a subcutaneous pocket. Pre-cross-linked hydrogel discs were implanted in these pockets, with 2 implants per mouse. The animals were euthanized at days 7, 14, and 21.

The self-healing property of the hydrogel was evaluated using a cut-and-heal test. Hydrogel discs were cut in half with a sharp blade, and the 2 pieces were brought into contact and allowed to heal at room temperature. After 15 min, the sample was lifted to confirm recovery of structural integrity.

### In vivo hemostasis assessment

The hemostatic efficacy of DTG bioglue was evaluated using 2 established in vivo models: (a) a rat abdominal aortic puncture model to assess vascular hemostasis and (b) a severe liver injury model to evaluate organ bleeding control. All procedures were conducted using male Sprague–Dawley rats (220 to 250 g). Animal experiments were approved by the Ethics Committee of Chonnam National University.

#### Liver injury model

Rats were anesthetized in an induction chamber using 2.5% to 3.5% sevoflurane, followed by intraperitoneal injection of ketamine and xylazine for general anesthesia. The upper abdominal area was shaved, and the body temperature was maintained above 35.9 °C using a heating pad. For light anesthesia, 1.5% to 2% sevoflurane with oxygen was administered via face mask. Once deep anesthesia was confirmed, rats were secured in a 30° inclined fixation device and draped. A midline laparotomy was performed, and the left lateral hepatic lobe was exposed using sterile cotton swabs. An abdominal incision was made to expose the liver, and a 5-mm-diameter injury was created using a dermal biopsy punch. Bioglue was immediately applied to the wound and cross-linked using visible light. A preweighed filter paper was placed beneath the liver to absorb blood and then weighed after 1 min to calculate blood loss. The abdominal wall and skin were closed using 3-0 Surgisorb sutures. At day 7 postoperation, rats were euthanized, and liver tissues from the wound sites were harvested, fixed in 4% paraformaldehyde, and sent to the Biomaterial R&BD Center at Chonnam National University for histological processing and hematoxylin-and-eosin staining.

For liver function analysis in rats, the hydrogel was applied and cured onto the liver surface following a sharp-needle puncture. Blood samples were collected from the tail vein on days 7 and 14 postimplantation. Serum alanine aminotransferase (ALT) levels were measured using the PicoSens ALT Assay Kit, following the manufacturer’s instructions.

#### Abdominal aortic puncture model

Rats were anesthetized using intraperitoneal injection of ketamine. Following a midline laparotomy, the abdominal aorta was exposed and temporarily occluded using 2 vascular clamps. A puncture wound was created using a fine-gauge needle. After allowing free bleeding for 5 s, the site was gently compressed with sterile gauze for 10 s, followed by wiping to remove excess blood. DTG bioglue was then applied immediately to the puncture site and cured using visible-light irradiation (handheld flashlight).

### Statistical analysis

All data were analyzed and plotted using GraphPad Prism (GraphPad Software, USA) and are presented as means ± standard deviation, unless otherwise stated. Statistical methods for each figure are described in the corresponding captions. A *P* value of <0.05 was considered statistically significant.

## Results and Discussion

**Fig. 2.**
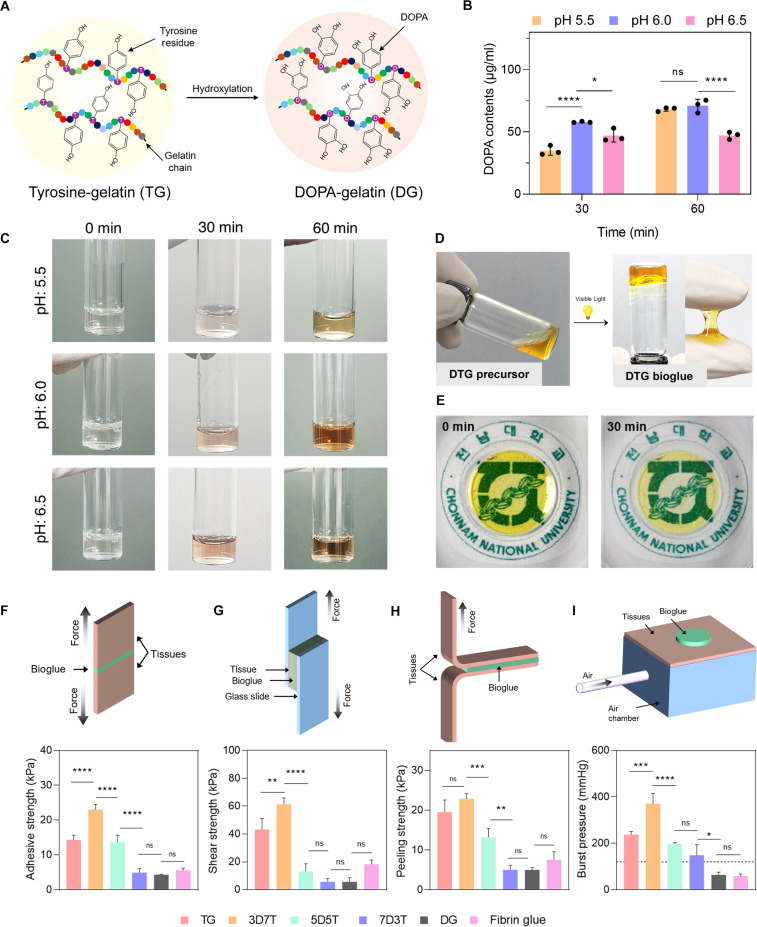
Optimization of 3,4-dihydroxy-l-phenylalanine (DOPA) functionalization and identification of the optimal DOPA–tyrosine gelatin (DTG) bioglue formulation. (A) Schematic of tyrosine hydroxylation within gelatin chains to generate DOPA. (B) Quantification of DOPA content (*n* = 3 per group; 2-way analysis of variance [ANOVA] with Tukey’s multiple comparison test; ns, not significant; *P < 0.05; ****P < 0.0001). (C) Representative images of gelatin solutions treated with tyrosinase for 0, 30, or 60 min. (D) Rapid gelation of the DTG precursor into adhesive DTG bioglue. (E) Representative images showing washout of residual photoinitiators after 30 min in phosphate-buffered saline (PBS). (F to I) Schematics and quantification of mechanical properties: (F) adhesive strength, (G) lap shear strength, (H) peel strength, and (I) burst pressure. The dashed line indicates arterial blood pressure (*n* = 3 per group; 1-way ANOVA with Tukey’s multiple comparison test; ns, not significant; *P < 0.05; **P < 0.01; ***P < 0.001; ****P < 0.0001).

Gelatin derived from porcine skin, known to be rich in tyrosine residues compared to other gelatin sources [30], was functionalized with DOPA through monophenol hydroxylation using tyrosinase. This approach was preferred to avoid the use of organic solvents or toxic reagents. Tyrosinase, a multifunctional copper-containing enzyme, catalyzes 2 sequential reactions: the hydroxylation of monophenols to *o*-diphenols (such as DOPA) (Fig. 2A) and the oxidation of *o*-diphenols to *o*-quinones [31].

Although tyrosinase exhibits optimal enzymatic activity at a pH range of 6.0 to 6.5 [32], we aimed to investigate the DOPA yield and its stability under varying pH conditions. This was important because under neutral to alkaline conditions, the likelihood of spontaneous oxidation increases, potentially compromising DOPA stability and leading to undesirable oxidation or premature cross-linking. Therefore, DOPA production was evaluated at pH 5.5, 6.0, and 6.5. The reaction was carried out for 60 min, and DOPA content was quantified at 30 and 60 min using Arnow’s assay, which specifically detects catechol-containing compounds. At 30 min, the highest DOPA concentration was observed at pH 6.0 (57.93 ± 0.44 μg/ml), followed by pH 6.5 (47.03 ± 4.23 μg/ml) and pH 5.5 (34.91 ± 3.11 μg/ml). These results suggest that at pH 5.5, tyrosinase activity was reduced because of the mildly acidic environment, whereas at pH 6.5, DOPA may have already begun to oxidize into DOPA-quinone, which is not detectable by Arnow’s assay. After 60 min, DOPA content further increased to 70.99 ± 4.27 μg/ml at pH 6.0 and 68.22 ± 1.29 μg/ml at pH 5.5, with no significant difference between them. However, at pH 6.5, no significant increase in DOPA concentration was observed, suggesting that an equilibrium might have been reached, where tyrosine is converted to DOPA and simultaneously oxidized to DOPA-quinone (Fig. 2B). A noticeable color change was observed during the reaction, with a brownish hue appearing at 30 min, which progressively darkened as the reaction continued (Fig. 2C). UV–visible spectroscopy of gelatin before incubation with tyrosinase revealed a characteristic absorption peak at 280 nm, attributable to aromatic amino acid residues [28]. Following incubation with tyrosinase, an increase in absorbance accompanied by spectral broadening in the 280- to 320-nm region was observed, consistent with the enzymatic hydroxylation of tyrosine residues to DOPA and the formation of characteristic catechol moieties (Fig. S3A).

After modification, DG was blended with TG in equal ratio and supplemented with Ru and SPS at concentrations of 0.5 and 5 mM, respectively, conditions previously identified as optimal. Upon light exposure, the DTG precursor rapidly transformed into a sticky hydrogel (Fig. 2D). Most of the remaining photoinitiators were removed during a subsequent PBS wash after 30 min (Fig. 2E). It is important to note that DOPA can also be formed during the dityrosine formation process from tyrosine in gelatin-based biomaterials, as Ru oxidizes the tyrosine, which reacts with hydroxide ion to yield DOPA as well [5]. Under the highly oxidative conditions of the reaction, partial oxidation of DOPA-modified gelatin cannot be ruled out. To investigate this, we measured the absorbance of hydrogel after curing and enzymatic digestion and observed an increase around 350 nm, indicative of oxidized catechol species (Fig. S3B). Despite this, our primary focus was to identify the optimal balance between the hydrogel components by varying their ratios and evaluating the resulting effects on adhesion.

Next, we sought to identify the optimal formulation of the DTG precursor by evaluating its adhesive performance through a series of mechanical tests on wet aortic tissue. DG and TG were blended in predefined ratios, and various formulations were screened. In tensile adhesive strength testing, the 3D7T (DOPA-rich and tyrosine-rich gelatin in a 3:7 ratio) formulation exhibited the highest performance, with an average adhesive strength of 23.08 ± 1.42 kPa, which was approximately 1.6 times greater than TG (14.33 ± 1.37 kPa) and 4.7 times higher than 7D3T. Notably, the DG formulation exhibited minimal strength, suggesting insufficient cohesion; however, physical entanglement of gelatin chains at room temperature may still contribute to the cohesion to an extent (Fig. 2F). Consistent with these findings, 3D7T also showed the highest shear strength, averaging 61.40 ± 4.54 kPa, indicative of an optimal balance of cohesion and adhesion (Fig. 2G). TG exhibited considerable shear resistance (43.28 ± 7.90 kPa), while 5D5T and 7D3T were markedly lower (13.20 ± 5.50 and 5.69 ± 2.23 kPa, respectively). DG and fibrin glue showed shear strengths of 5.70 ± 2.93 and 18.33 ± 3.07 kPa, respectively. In terms of peel-strength test (Fig. 2H), 3D7T again showed superior performance over the other formulations and fibrin glue; however, the difference was not significant when compared to DG. Burst-pressure testing was conducted using a custom-built setup equipped with manometric pressure monitoring (Fig. 2I and Movie S1), further demonstrating the superior sealing capability of 3D7T, which withstood an average pressure of 371.33 ± 35.78 mmHg, making 3D7T considerably stronger than clinically used fibrin glue. The burst pressure of 3D7T was notably higher than the average physiological arterial blood pressure (~120 mmHg).

The superior performance of 3D7T across all tests can be attributed to its optimized combination of cohesive and adhesive mechanisms. DOPA groups contribute to strong interfacial adhesion through hydrogen bonding, metal chelation, and π–π interactions with tissue surfaces [33,34]. Simultaneously, unmodified tyrosine residues from TG promote effective dityrosine cross-linking, reinforcing the bulk cohesion of the hydrogel. In contrast, TG alone, although capable of exhibiting maximum dityrosine crosslinks due to maximum tyrosine availability, may lack sufficient generation of DOPA, resulting in weaker surface adhesion. Meanwhile, formulations such as 5D5T, 7D3T, and DG, despite having a higher DOPA content, likely suffer from insufficient cohesion due to reduced tyrosine availability for cross-linking. This observation may also be explained by the fact that during the Ru/SPS-based photocrosslinking process, the oxidative environment may readily oxidize DOPA into quinone in formulations with lower tyrosine content and increased DOPA levels. This undesirable oxidation may result in simultaneous loss of both cohesion and adhesion leading to poor structural stability as well as diminished surface-level adhesion. Therefore, maintaining an optimal balance between available tyrosine and DOPA is critical. Taken together, these findings identify 3D7T as the optimal formulation, offering the optimal balance between gel cohesion and tissue adhesion. This formulation was thus designated as DTG bioglue and used in subsequent experiments. The DTG bioglue demonstrated superior mechanical performance relative to fibrin glue, indicating that the material develops sufficient robustness during early curing to withstand physiological pressures relevant to surgical hemostasis.

**Fig. 3.**
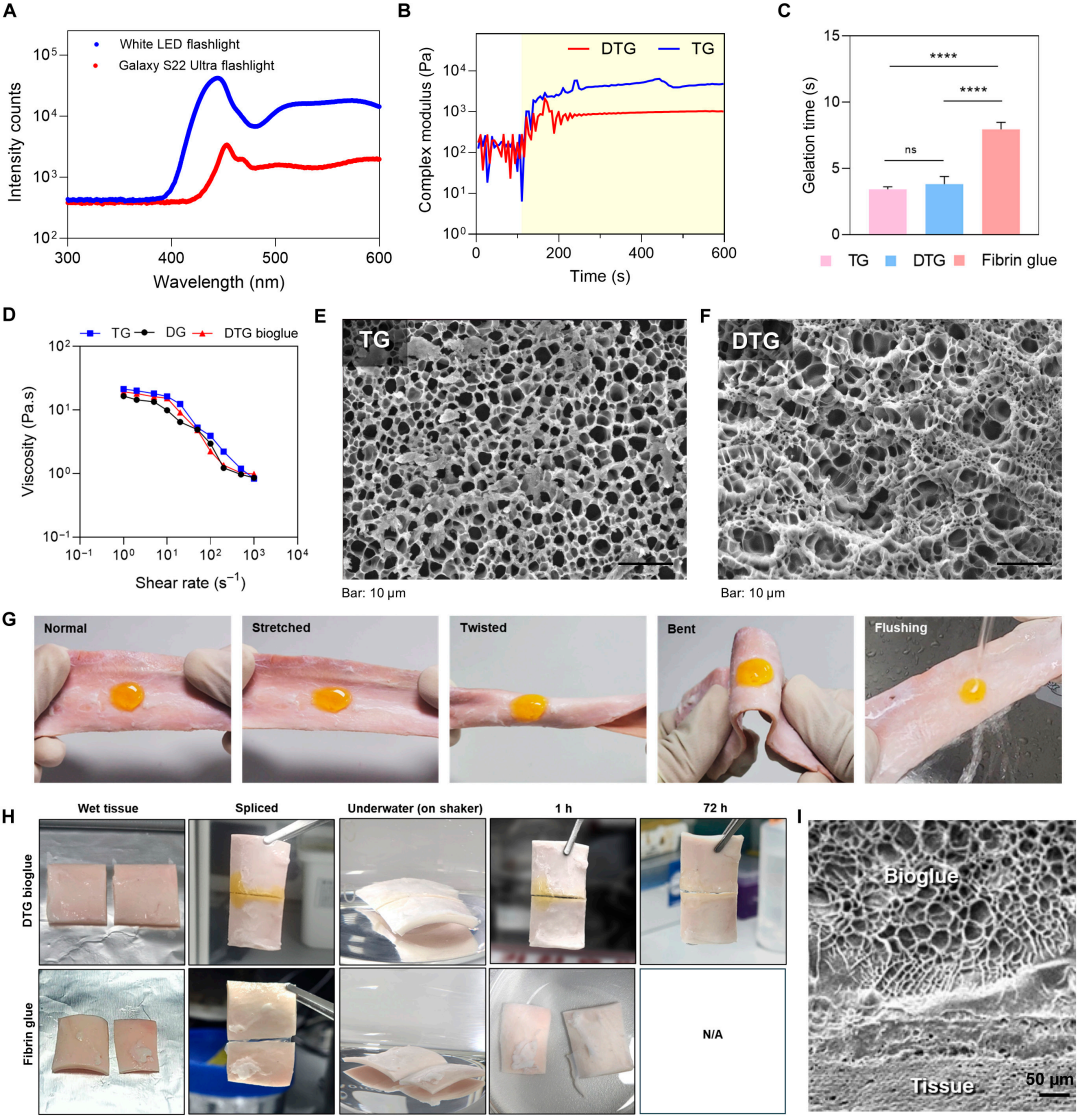
Rheological, structural, and underwater adhesion properties of 3,4-dihydroxy-l-phenylalanine (DOPA)–tyrosine gelatin (DTG) bioglue. (A) Emission spectra of light sources including a white light-emitting-diode (LED) flashlight and Galaxy S22 Ultra. (B) Phase transition behavior of DTG bioglue and TG hydrogel. Yellow shaded area indicates that the light was turned on during this period. (C) Gelation kinetics of TG, DTG, and fibrin glue (*n* = 3 per group; 1-way ANOVA with Tukey’s multiple comparison test; ns, not significant; ****P < 0.0001). (D) Shear-thinning behavior of the hydrogels, shown by viscosity changes under increasing shear rate. (E and F) Scanning electron microscopy (SEM) images of (E) TG and (F) DTG bioglue, highlighting porous microstructures. (G) Adhesion of DTG bioglue to wet porcine aorta under stretching, twisting, bending, and water flushing. (H) Representative images of tissue adhesion using fibrin glue versus DTG bioglue under submerged conditions on a rocker for up to 72 h N/A, not applicable.. (I) SEM image of the tissue–DTG bioglue interface, showing ultrastructural adhesion.

The phase transition of the DTG precursor into DTG bioglue upon light exposure was assessed using a photorheometer by monitoring the change in complex modulus over time under light. A handheld white LED flashlight was characterized for its emission spectrum, which revealed a peak intensity at approximately 450 nm within the visible-light range. This wavelength matches the activation requirements of Ru-based photocrosslinking systems, making this common LED suitable for initiating hydrogel cross-linking. In addition, the emission spectrum of a smartphone flashlight was measured, which also exhibited a peak near 450 nm, suggesting that multiple common light sources could serve as effective cross-linking initiators (Fig. 3A). Furthermore, the irradiance profile of the LED flashlight was characterized across its emission range, and the integrated irradiance between 400 and 600 nm was quantified at varying distances as well. Upon light irradiation, complex modulus increased sharply and reached a plateau within seconds, suggesting saturation of dityrosine cross-links (Fig. 3B). This immediate rise and stabilization of modulus confirm that the DTG formulation retains its photocrosslinking ability even after blending DOPA-functionalized and unmodified gelatin components. Notably, the rapid gelation kinetics of DTG bioglue outperformed the gelation time of fibrin glue (Fig. 3C). For translational application, both curing kinetics and early-stage mechanical integrity are critical parameters for hemostatic adhesives. Our system utilizes low-intensity white light (5 mW/cm^2^), enabling safe and practical activation with portable clinical LED devices. The adhesive exhibits rapid gelation within 5 to 10 s and achieves full mechanical stabilization within 1 min. This curing profile compares favorably to clinically used fibrin glue, which typically requires tens of seconds for gelation and longer for mechanical strengthening (Table S1). Further rheological analysis was conducted to examine the flow behavior of TG, DG, and DTG bioglue formulations by measuring viscosity across a range of shear rates (Fig. 3D). All formulations demonstrated characteristic shear-thinning behavior, as shown by a continuous decline in viscosity with increasing shear rate, an important feature for applications such as injectable therapeutics and microextrusion-based bioprinting, where ease of delivery and shape retention are critical. Microstructural morphology of TG and DTG hydrogels were observed under SEM (Fig. 3E and F). Both formulations exhibited a porous, interconnected network structure typical of gelatin-based hydrogels. However, distinct differences in pore size distribution and network density were observed.

The DTG bioglue further demonstrated strong and stable adhesion to porcine skin under physical deformation. The cured bioglue retained its adherence under various mechanical deformations such as stretching, twisting, and bending. Notably, even under dynamic conditions like water flushing, the hydrogel remained firmly anchored to the tissue surface (Fig. 3G and Movie S2). The DTG hydrogel also exhibited a self-healing property as confirmed by a cut-and-heal test (Fig. S4A).

To assess the stability of adhesion under dynamic underwater conditions, we carried out an ex vivo assessment in which 2 segments of porcine aorta were joined edge-to-edge using the respective bioadhesives and submerged in water and PBS within a container placed on a shaking platform for 72 h. The tissues bonded with DTG bioglue maintained stable adhesion throughout the entire duration (Fig. 3H and Fig. S4B). In contrast, samples joined with fibrin glue detached within the first hour. SEM analysis further confirmed that the DTG bioglue formed a continuous interface with the tissue surface (Fig. 3I).

**Fig. 4.**
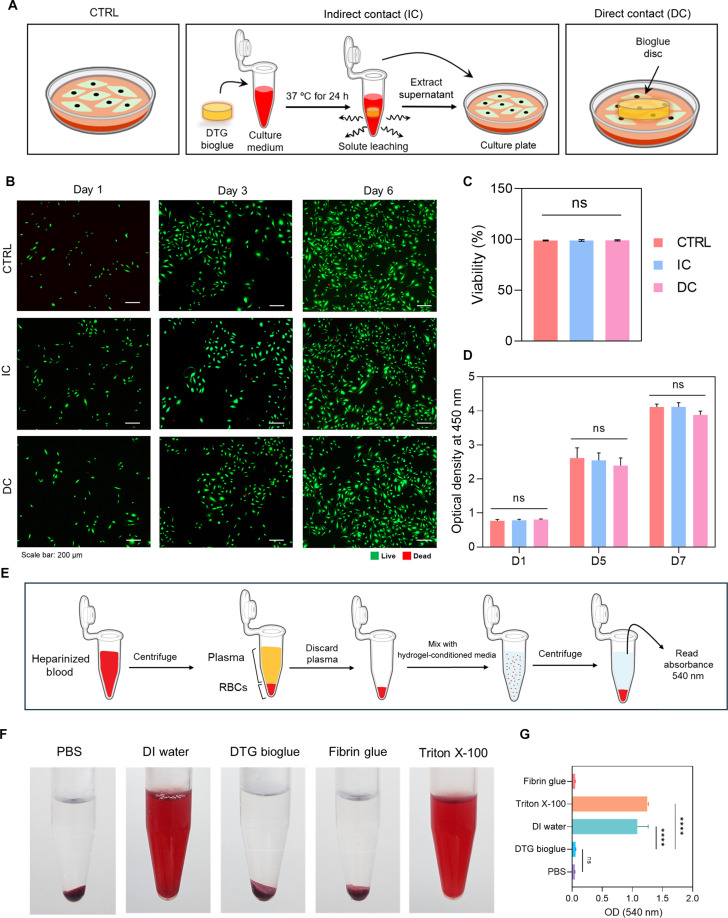
Cytocompatibility and hemocompatibility of 3,4-dihydroxy-l-phenylalanine (DOPA)–tyrosine gelatin (DTG) bioglue. (A) Schematic of the cytocompatibility test performed under indirect contact (IC) and direct contact (DC) conditions. (B) Live/dead fluorescence images of human umbilical vein endothelial cells cultured under control (CTRL), IC, and DC conditions over time. (C and D) Quantification of (C) cell viability and (D) metabolic activity using the Cell Counting Kit-8 (CCK-8) assay [*n* = 3; 1-way ANOVA (C) and 2-way ANOVA with Tukey’s multiple comparison test (D); ns, not significant]. (E) Schematic of hemocompatibility testing to assess interactions of DTG bioglue with red blood cells (RBCs). (F) Representative images of hemoglobin release into the supernatant. DI, deionized. (G) Hemolysis ratio quantified from the supernatant (*n* = 3; 1-way ANOVA with Tukey’s multiple comparison test; ns, not significant; ****P < 0.0001). OD, optical density.

The cytocompatibility of the DTG bioglue was assessed using both direct and indirect contact culture modes using HUVECs (Fig. 4A). Under both conditions, the cells exhibited high viability (>95%), which was not significantly different from those cultured on a culture plate (Fig. 4B and C). To further evaluate the effect of the bioglue on cell proliferation, we measured metabolic activity using a CCK-8 assay on days 1, 5, and 7 (Fig. 4D). Across the entire culture period, HUVECs maintained comparable metabolic activity in both contact modes, with no statistically significant differences observed between the DTG-bioglue-treated groups and control, which indicate that the DTG bioglue is highly cytocompatible and does not negatively impact cell viability or metabolic function. Moreover, the endothelial monolayer exhibited a representative cobblestone morphology after 14 d of exposure to the DTG hydrogel, with CD31 localized at cell–cell junctions (Fig. S5).

The blood compatibility of the DTG bioglue was assessed using a hemolysis assay to evaluate its potential to cause RBC damage. The RBC suspension was incubated with hydrogel-conditioned solutions, followed by centrifugation to separate intact RBCs from released hemoglobin (Fig. 4E). The supernatants were then subjected to spectrophotometric analysis to quantify hemolysis. Deionized water and Triton X-100 were used as positive controls, known to induce hemolysis [35], while PBS served as a negative control. Fibrin glue was also included for comparison. Visual inspection revealed that, except for the positive controls (deionized water and Triton X-100), the supernatants from all other groups, including DTG bioglue, remained clear, indicating minimal hemoglobin release (Fig. 4F). Quantitative spectrophotometric analysis confirmed this observation: The DTG bioglue exhibited a comparable optical density to that of the PBS group and significantly lower than that of the positive controls (Fig. 4G). These results demonstrate that the DTG bioglue is hemocompatible and suitable for applications involving direct blood contact.

To assess in vivo biodegradability, we subcutaneously implanted the hydrogel on the dorsal side of mice. At 7 d postimplantation, the hydrogel disc remained largely intact and well defined within the subcutaneous pocket. By 14 d, subtle remodeling of the implant site was observed, and by 21 d, degradation was most pronounced, as indicated by a substantial reduction in the original hydrogel volume at the implant site (Fig. S6A). In vitro characterization of the hydrogel further elucidated its degradation and swelling dynamics, providing insight into its expected in vivo behavior. When incubated in PBS, the hydrogel retained approximately 90% of its initial mass even after 40 h, indicating high structural stability under nonenzymatic conditions. In contrast, exposure to collagenase induced rapid enzymatic degradation: Mass loss accelerated noticeably after ~10 to 15 h, with near-complete breakdown observed by 40 h (Fig. S6B). Collagenase, used as a model enzyme, provides a standardized in vitro assessment of hydrogel degradability, which, although faster than in vivo, demonstrates that the hydrogel maintains initial mechanical integrity for effective sealing while undergoing gradual degradation over 3 weeks in vivo. Swelling kinetics demonstrated the highly hydrophilic nature of the network, with rapid and substantial water uptake reaching a plateau of ~400% to 500% within the first 10 to 20 h and became stable afterwards (Fig. S6C).

**Fig. 5.**
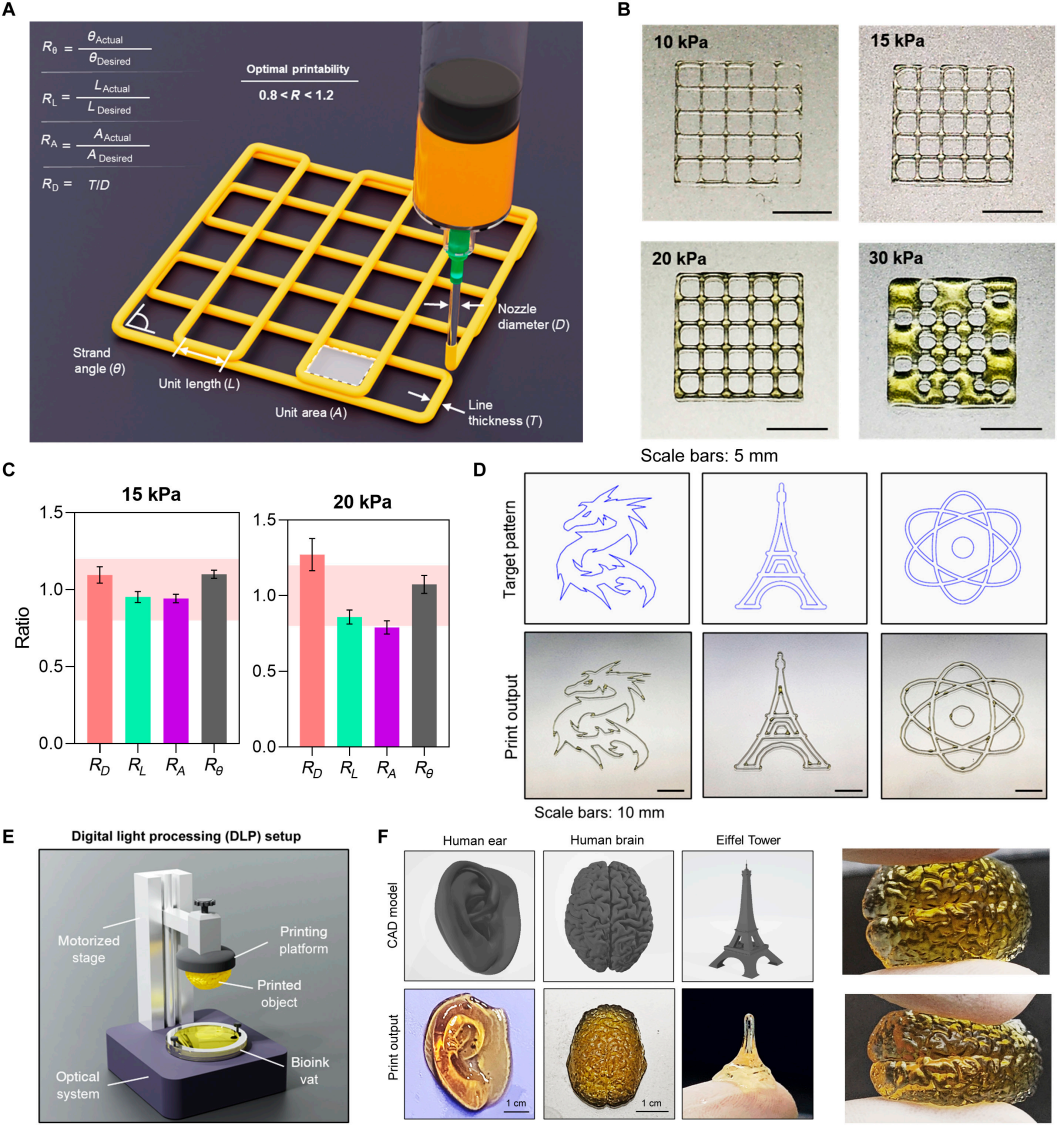
Printability assessment using 3,4-dihydroxy-l-phenylalanine (DOPA)–tyrosine gelatin (DTG) as bioink. (A) Schematic illustration of matrices used to evaluate 2-dimensional (2D) shape fidelity. (B) Representative images showing printed structures at varying extrusion pressures. (C) Quantification of 2D shape fidelity evaluation matrices at 15 and 20 kPa. (D) Demonstration of various printed patterns, including a dragon, Eiffel Tower, and atomic structure, under optimized printing conditions. (E) Digital light processing (DLP) setup used for volumetric bioprinting. (F) Representative images of computer-aided design (CAD) models and the corresponding structures fabricated using the DLP system. CAD models for printing were sourced from NIH 3D (3d.nih.gov) and Free3D (free3d.com).

To evaluate the printability of the DTG bioglue, we explored its suitability for 3D printing using both microextrusion and DLP setups, considering its shear-thinning behavior and rapid photocrosslinking capability. Initially, extrusion-based print fidelity was assessed at varying pneumatic pressures to determine optimal printing conditions. Printing accuracy was quantified by comparing the matrices, such as line width, unit length, unit area, and line angle, between the printed constructs and the intended design. A dimensional fidelity ratio between 0.8 and 1.2 was defined as acceptable range of deviation from the intended design (Fig. 5A).

As the printing pressure was increased from 10 to 30 kPa, structures printed at 15 and 20 kPa demonstrated good fidelity. In contrast, 10 kPa resulted in underextrusion, while 30 kPa led to overextrusion and loss of detail (Fig. 5B). At 15 kPa, all measured matrices remained within the acceptable range, confirming this as optimal pressure for printing (Fig. 5C). Various shapes, including a dragon, atomic model, and Eiffel Tower, were patterned as representative printed structures (Fig. 5D). Moreover, using a custom-built DLP setup (Fig. 5E), we successfully fabricated volumetric tissue constructs, including models of a human ear, Eiffel Tower, and human brain. These printed structures demonstrated excellent mechanical integrity, further confirming the suitability of the DTG bioglue for high-resolution, light-based 3D bioprinting (Fig. 5F).

We further demonstrated the feasibility of engineered tissue assembly using a bioglue-assisted approach. Conventionally, hybrid bioprinting, which alternates the deposition of polymeric frameworks and bioinks, has been used to fabricate centimeter-scale tissue constructs [36–38]. However, this approach is limited by the prolonged printing duration, particularly when fabricating larger tissue constructs on the scale of a few centimeters, which is inherent to microextrusion-based techniques. Such extended printing sessions can lead to decreased cell viability and may also result in heterogeneous cell distribution, especially when using low-viscosity bioinks, due to rapid cell sedimentation under gravity during the printing process.

**Fig. 6.**
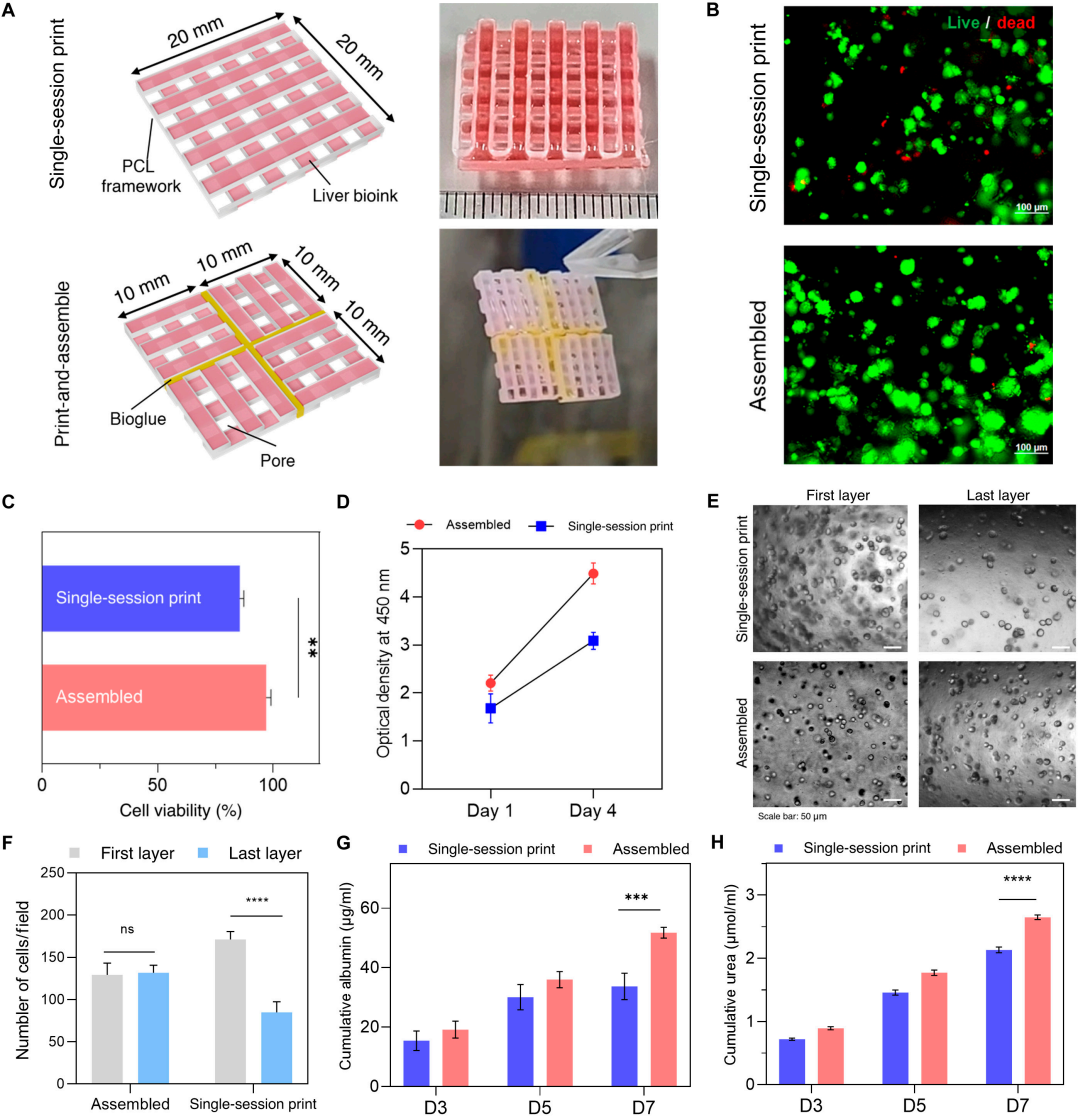
Proof of concept for engineered tissue assembly using 3,4-dihydroxy-l-phenylalanine (DOPA)–tyrosine gelatin (DTG) bioglue. (A) Schematic and gross images of structures fabricated via single-session printing and print-and-assemble strategies. PCL, polycaprolactone. (B) Live/dead fluorescence images of HepG2 cells at day 7 using both approaches. (C) Cell viability quantified on day 7 (*n* = 3; 2-tailed *t* test **P < 0.01). (D) Metabolic activity assessed by Cell Counting Kit-8 (CCK-8) assay on days 1 and 4. (E) Representative images of cell density distribution in the first and last printed layers. (F) Quantification of cell number per field in first and last layers (*n* = 3 per group; 2-way ANOVA with Tukey’s multiple comparison test; ns, not significant; ****P < 0.0001). (G and H) Functional assessment of HepG2 cells: (G) cumulative albumin secretion and (H) cumulative urea secretion on days 3, 5, and 7 (*n* = 3; 2-way ANOVA with Tukey’s multiple comparison test; ns, not significant; ***P < 0.001; ****P < 0.0001).

To overcome these limitations, we hypothesized that assembling smaller constructs, each printed within an acceptable time window, using our DTG bioglue could enable the fabrication of larger constructs, with minimal effect on tissue functionality. As a proof of concept, we aimed to surpass the previously reported 1 cm × 1 cm liver tissue model [39] created via hybrid bioprinting by printing a 2 cm × 2 cm construct using modular assembly. We compared 2 fabrication strategies: a single-session direct print versus a bioglue-assisted modular assembly. In the single-session printing approach, the entire 2 cm × 2 cm construct was printed continuously from a single bioink cartridge over approximately 40 min. In the modular assembly approach, 4 individual 1 cm × 1 cm units were each printed separately using a freshly loaded cartridge, requiring only ~10 min per unit. The individually printed modules were then assembled into the target 2 cm × 2 cm construct using DTG bioglue. This strategy was designed to minimize cell sedimentation within the cartridge during printing, as each unit is fabricated within a short time window before significant gravitational settling can occur (Fig. 6A). Cell viability assessments on day 7 revealed that the assembled construct maintained high viability (>95%; Fig. 6B and C), whereas the single-session printed construct showed reasonable but significantly lower viability (~85%) compared to the assembled construct. CCK-8 assay results confirmed reduced metabolic activity in the single-session printed tissues (Fig. 6D). Furthermore, analysis of cell density distribution across the first and last printed layers revealed a stark gradient in the single-session print, whereas the assembled construct exhibited uniform cell density throughout (Fig. 6E and F). The difference in cell distribution could be attributed to the viscosity of the bioink, which allows cells to gradually settle under gravity during printing. Since the first layer of bioink is deposited after the framework is printed, the cells start to settle, resulting in a higher cell density in the first layer. In contrast, the second layer contains relatively lower density compared to the first.

Liver-specific functionality was evaluated by quantifying urea and albumin secretion. Consistent with the viability and proliferation results, the single-session printed tissues exhibited significantly lower levels of both compared to the assembled structures by day 7 (Fig. 6G and H), highlighting the superior functional performance of the bioglue-assisted assembly approach.

**Fig. 7.**
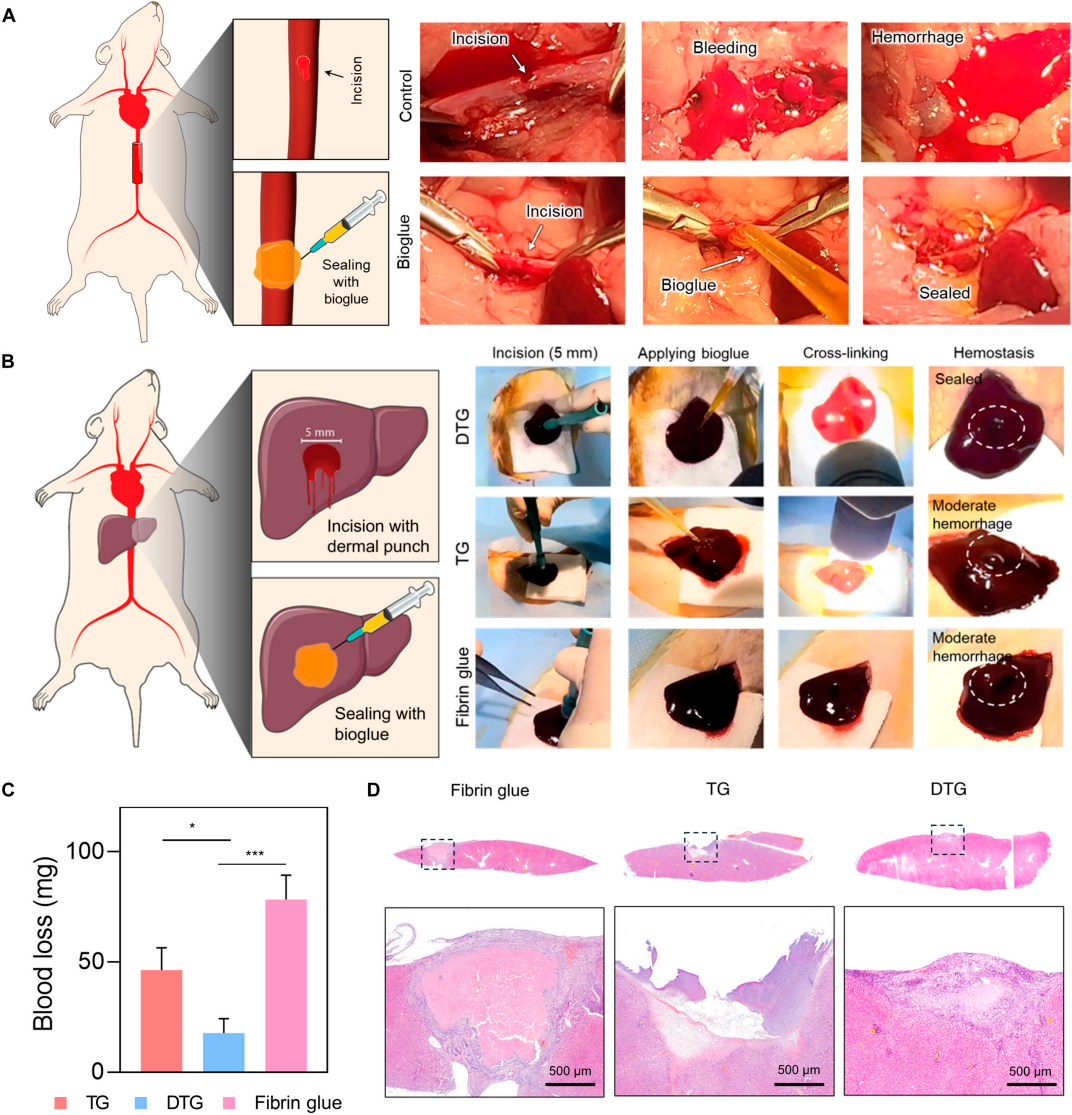
In vivo hemostatic performance of 3,4-dihydroxy-l-phenylalanine (DOPA)–tyrosine gelatin (DTG) bioglue. (A) Rat abdominal aorta hemorrhage model used to evaluate the sealing efficacy. DTG bioglue effectively sealed the incision site after clamp removal, while controls exhibited severe bleeding. (B) Severe liver injury model made using a 5-mm dermal punch. DTG bioglue rapidly sealed the wound, outperforming fibrin and unmodified gelatin. (C) Quantification of total blood loss after bioglue application (*n* = 3; 1-way ANOVA with Tukey’s multiple comparison test; *P < 0.05, ***P < 0.001). (D) Hematoxylin-and-eosin-stained histological images of the injury site on day 7.

To evaluate the hemostatic performance of the DTG bioglue, we first tested it using a rat abdominal aorta injury model simulating massive hemorrhage. Upon removal of vascular clamps after applying the bioglue at the defect made with a sharp needle, the DTG bioglue significantly reduced blood loss compared to control, which exhibited profuse bleeding (Fig. 7A). The enhanced hemostatic effect of the DTG bioglue can be attributed to its rapid in situ photocrosslinking and robust wet-tissue adhesion, which facilitated strong adherence to the wound surface and the formation of a stable hydrogel barrier.

Due to its rich blood supply, delicate tissue structure, and fragile outer capsule, the liver is among the most frequently damaged organs in abdominal trauma [40]. A liver hemorrhage model was used to assess its sealing capability under wet-tissue-surface condition. A 5-mm circular incision was made using a biopsy punch, and bioglue formulations were applied to the wound area. The DTG bioglue achieved complete sealing within 30 s of light irradiation, with no observable bleeding (Movie S3). In contrast, both TG (Movie S4) and fibrin glue (Movie S5) exhibited moderate hemorrhage at the wound site (Fig. 7B). Quantitative blood loss measurements further confirmed DTG’s superior hemostatic effect, approximately 4 times less blood loss than fibrin glue (Fig. 7C).

Histological analysis of the sealed liver tissues 1 week postapplication revealed that the TG bioglue resulted in hydrogel displacement from the wound site. Fibrin glue showed the formation of a relatively large blood clot, whereas the DTG bioglue exhibited a comparatively smaller area of clotting. All groups exhibited a considerable infiltration of inflammatory cells (Fig. 7D). These findings highlight DTG bioglue’s promising potential for rapid and effective bleeding control under severe injury conditions.

Liver function is commonly assessed by measuring serum levels of ALT, a cytosolic enzyme released into the bloodstream upon hepatocellular injury. Monitoring ALT provides a sensitive indicator of liver damage or stress and is routinely used in preclinical studies to evaluate the biocompatibility of implanted materials. In the context of hydrogel implantation following liver injury, transient elevations in ALT are expected as part of the normal tissue response to surgical trauma. However, persistent or excessive increases may indicate hepatotoxicity or adverse effects of the implanted material. Therefore, we measured serum ALT levels for 2 weeks postimplantation of hydrogel on liver following a needle-induced puncture. Measurements were taken at day 7 and day 14 postimplantation. On day 7, ALT levels were slightly elevated (~83 U/l), likely reflecting the natural hepatic response to the surgical injury. By day 14, ALT levels returned to the normal range (~50 U/l) [41], indicating recovery of liver function (Fig. S6D).

## Conclusion

Taken together, we developed and thoroughly characterized the visible-light-curable DTG bioglue, formulated via monophenol hydroxylation of porcine-derived gelatin. By leveraging tyrosinase-mediated DOPA functionalization under controlled pH conditions, we used a simple yet effective strategy to introduce catechol groups without relying on harsh chemical conjugation. The optimized formulation, blending DOPA-rich and tyrosine-rich gelatin in a 3:7 ratio (3D7T), offered a synergistic balance between interfacial adhesion and cohesive strength, enabling rapid gelation, enhanced mechanical stability, and strong tissue adhesion under wet conditions.

Mechanically, DTG outperformed conventional sealants including fibrin glue and unmodified gelatin across multiple tests: tensile, shear, peel, and burst pressure. Its shear-thinning behavior and fast visible-light-induced photocrosslinking also make it particularly compatible with both extrusion-based and DLP-based bioprinting platforms, enabling high-fidelity printing of complex patterns and volumetric constructs. Moreover, DTG’s cytocompatibility and hemocompatibility were confirmed through both direct and indirect contact assays with HUVECs and RBCs, respectively, validating its biosafety for clinical use. While rapid hemostasis and mechanical sealing are primary goals, surgical adhesives can be enhanced with bioactive functions to improve outcomes. Our visible-light-cross-linked network can incorporate bioactive molecules without compromising curing; for example, basic fibroblast growth factor bFGF has been successfully loaded into Ru–SPS and gelatin-based adhesives in a previous study [42]. We anticipate that similar bioactive compounds could be integrated to further optimize our system for future clinical applications. Recent research has also focused on functionalizing existing adhesive hydrogel systems by incorporating conductive or stimuli-responsive features to provide sensory feedback, as exemplified by multifunctional conductive structural color hydrogels for interactive electronic interfaces [43]. In parallel, antibacterial modifications have been explored to prevent infections in dynamic wound environments [44].

A novel application demonstrated in this study is the assembly of engineered tissues, where DTG was used to construct modular liver tissue units with improved cell viability, uniform cell distribution, and enhanced functionality compared to single-session bioprinting. Although the long-term maintenance of adhesion in vivo and the fabrication of complex design structures remain to be investigated, our proof-of-concept study could pave the way for volumetric tissue construction using a microextrusion approach. Furthermore, in vivo validation using rat models of abdominal aortic hemorrhage and severe liver injury confirmed the DTG bioglue’s rapid hemostatic sealing capability, with significantly reduced blood loss and evidence of early cellular infiltration and tissue repair at the wound site. Future studies will focus on evaluating the long-term in vivo biocompatibility and immune responses to DTG in large animal models. To expand its therapeutic potential, DTG could be functionalized with bioactive agents such as angiogenic factors (e.g., vascular endothelial growth factor), antimicrobial peptides, or immunomodulatory compounds, enabling broader application in wound healing, regenerative medicine, and infection-prone surgical contexts. Although visible-light-induced curing provides precise spatial and temporal control, integrating secondary enzymatic cross-linking of gelatin may improve mechanical robustness and durability.

## Data Availability

Supporting data are available from the authors upon reasonable request.

## Supplementary Materials

Supplementary 1 Figs. S1 to S6 Table S1 Movies S1 to S5 References [45–50]
