# Supplementary material for: Visible-Light-Curable Catechol-Reinforced Gelatin Bioglue for Rapid Hemostasis and Engineered Tissue Assembly
Source: Biomater Res. 2026 May 7;30:0360. doi: 10.34133/bmr.0360 (PMC13150077; doi:10.34133/bmr.0360)
Supplement: Supplementary 1 — Figs. S1 to S6 Table S1 Movies S1 to S5 References [45–50] [file bmr.0360.f1.zip › Supplementary Information_revised.docx]

**Supplementary Information**

**Visible Light-Curable Catechol-Reinforced Gelatin Bioglue for Rapid Hemostasis and Engineered Tissue Assembly**

*Ashfaq Ahmad ^1,2^, Jian Shin ^1^, Jaylord M. Pioquinto ^3^, Se Eun Kim ^3^,* *Yong Sook Kim ^4,5^, Jessie S. Jeon^6^, Yeong-Jin Choi ^7,8,*^ and Hee-Gyeong Yi ^1,2,9,*^*

1 Department of Convergence Biosystems Engineering, College of Agriculture and Life Sciences , Chonnam National University, Gwangju, Republic of Korea

2 Interdisciplinary Program in IT-Bio Convergence System, Chonnam National University

3 Department of Veterinary Surgery, College of Veterinary Medicine, Chonnam National University, Gwangju, Republic of Korea

4 Biomedical Research Institute, Chonnam National University Hospital, Gwangju, Republic of Korea

5 Medical Research Center, Chonnam National University Medical School, Hwasun, Republic of Korea

6 Department of Mechanical Engineering, Korea Advanced Institute of Science and Technology (KAIST)

7 Advanced Bio and Healthcare Materials Research Division, Korea Institute of Materials Science (KIMS), Changwon, Republic of Korea

8 Advanced Materials Engineering, Korea National University of Science and Technology (UST), Daejeon, Republic of Korea

9 Institute for Biomedical Science, Chonnam National University Hospital Hwasun, Hwasun, Republic of Korea

*Co-corresponding authors: Yeong-Jin Choi (jinchoi@kims.re.kr) and Hee-Gyeong Yi ([hgyi@chonnam.ac.kr](mailto:hgyi@chonnam.ac.kr))

**This supporting information includes the supplementary movies below.**

Movie S1. Burst pressure assessment of bioglue-mounted tissue under pressurization.

Movie S2. Adhesion performance of bioglue on porcine aorta under running tap water.

Movie S3. Sealing of liver hemorrhage using DTG bioglue.

Movie S4. Sealing of liver hemorrhage using TG hydrogel.

Movie S5. Sealing of liver hemorrhage using fibrin glue.


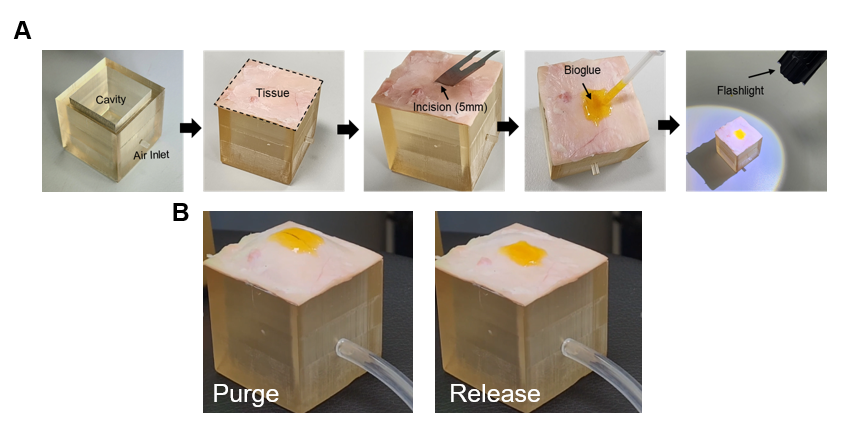


Figure S1. Customized setup for burst pressure assessment. A) Steps of procedure: a 3D-printed air chamber with an open-top cavity and air inlet is covered with tissue, followed by a 5 mm incision, application of bioglue, and curing using visible light. B) bioglue under purge and release state.


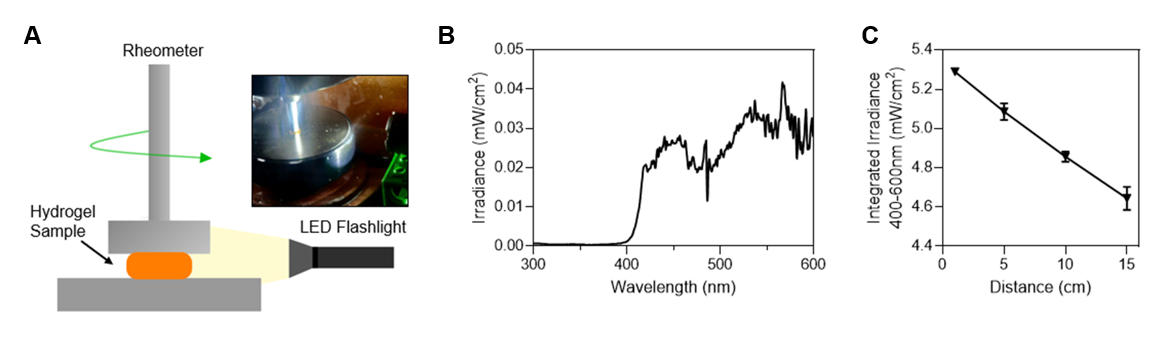


Figure S2. (A) Customized rheometer setup for exposing the sample to LED light during time-sweep measurements. (B) Irradiance spectrum of the LED light measured from 300 nm to 600 nm. (C) Profile of integrated irradiance as a function of distance from the LED source.


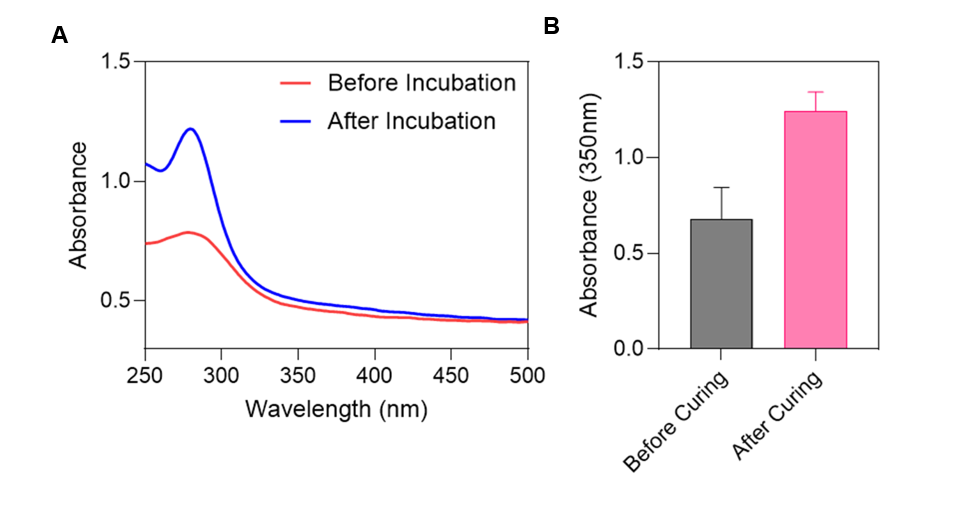


Figure S3. (A) UV–visible absorption spectra of gelatin solutions containing tyrosinase, recorded before and after incubation at 37 °C. (B) Absorbance at 350 nm, used to monitor DOPA oxidation during the curing process.


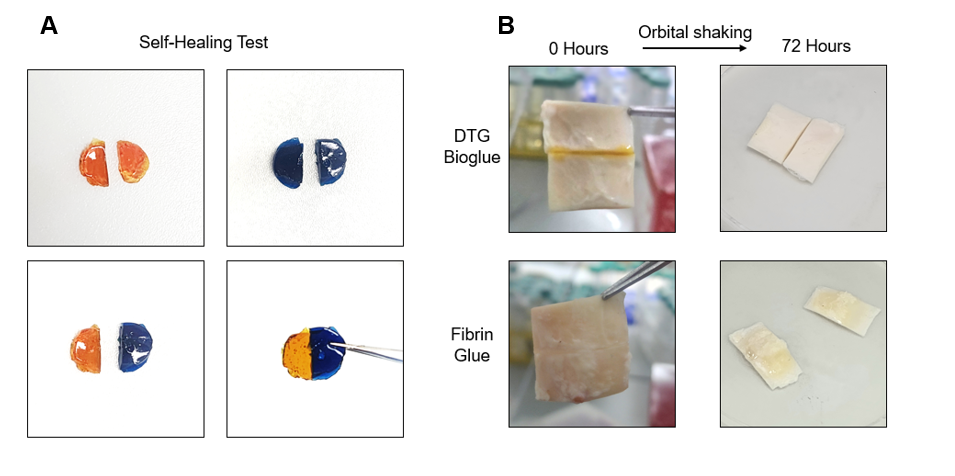


Figure S4. (A) Cut-and-heal test to assess the self-healing property of the hydrogel. (B) Qualitative evaluation of the adhesive performance of DTG and fibrin glue in joining two aortic tissues, with samples submerged in PBS under continuous orbital shaking.


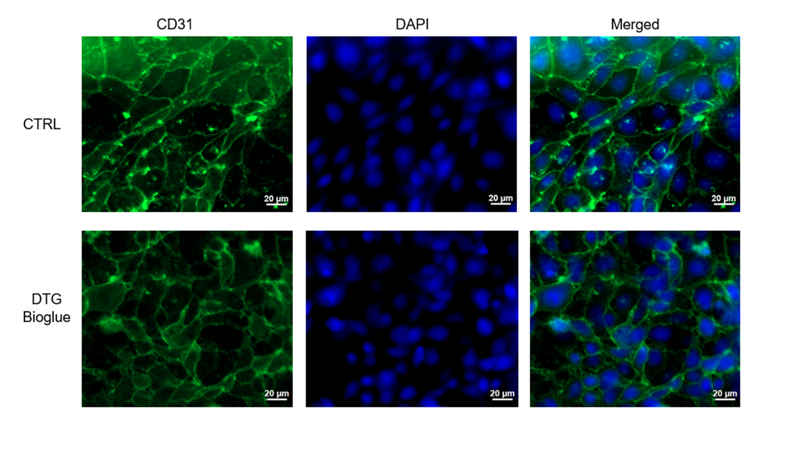


Figure S5. Immunostaining of endothelial cell monolayers for CD31 (green) and DAPI (blue) at Day 14 following exposure to the bioglue.


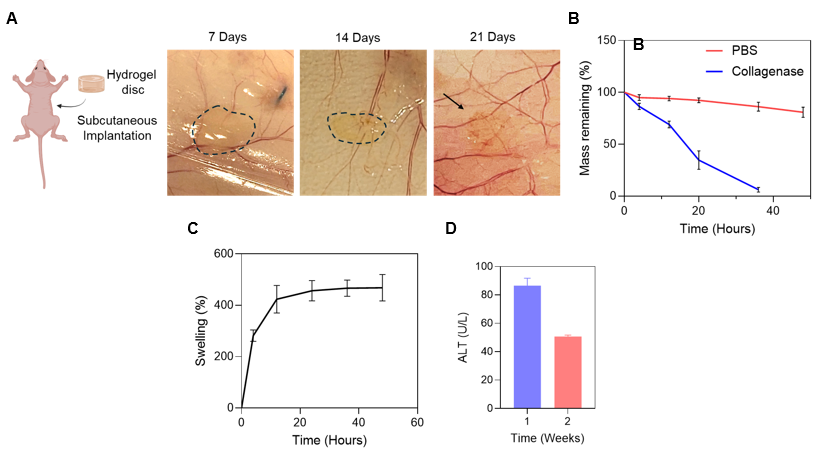


Figure S6. Evaluation of DTG bioglue degradation and performance. A) Photographs showing hydrogel degradation at weekly intervals over 3 weeks following subcutaneous implantation in mice. B) In vitro degradation profile of DTG bioglue in PBS and collagenase solutions. C) Swelling behavior of DTG bioglue over time. D) Serum ALT levels in rats at 1 and 2 weeks after application of the hydrogel on punctured livers.

Table S1: Pros and cons of existing and DTG bioglue system.

| **Bioglue**  **System** | **Working**  **Mechanism** | **Curing Time** | **Pros** | **Cons** | **Reference** |
| --- | --- | --- | --- | --- | --- |
| Fibrin Glue (Tisseel®, Evicel®) | Thrombin-mediated fibrinogen → fibrin conversion | 10–60 s | FDA-approved; mimics natural coagulation  Excellent biocompatibility and biodegradability  Minimal foreign body response. | Weak wet adhesion; easily displaced by blood  Poor mechanical strength  Viral transmission risk; expensive | [45] |
| Cyanoacrylate Glue (Dermabond®) | Anionic polymerization on contact with moisture/blood | 2.5 min | Rapid and strong bonding  High hemostatic rate (87–93%)  Good shelf stability | Exothermic curing; tissue burns  Toxic degradation products (formaldehyde)  Brittle; inflammatory; possibly carcinogenic | [46] |
| GelMA-based Hydrogels | UV/visible light photo-initiated radical polymerization | 5–20 s (visible light/LAP)  ≥10 s (UV/Irgacure)  42–300 s (riboflavin) | ECM-mimetic; tunable mechanical properties  Controllable gelation via light exposure  Good biocompatibility | UV may damage tissue; limited penetration  Photoinitiator cytotoxicity  Insufficient wet adhesion alone; swelling issues | [47] |
| PEG-based Hydrogels (DuraSeal®) | NHS-ester/amine reaction or Michael addition | ~ 1 min | Highly tunable; rapid gelation  Low immunogenicity and FDA-approved variants  Excellent hydrophilicity | Excessive swelling  Weak tissue adhesion without functionalization | [48] |
| Chitosan-based Hydrogels | Schiff base, ionic, genipin, or thermal crosslinking | <30 s (Schiff base) | Inherent hemostatic and antibacterial activity  Biodegradable; abundant; low cost  Injectable; fills irregular wounds | Poor solubility at physiological pH  Weak mechanical properties alone  Requires modification for wet adhesion | [49] |
| Catechol/DOPA-functionalized Hydrogels | Oxidative (NaIO₄, Fe³⁺), enzymatic (HRP/H₂O₂), or Michael addition | <20 s (NaIO₄, pH 8)  1–6 min (lower pH) | Superior wet adhesion via catechol–tissue bonds  Versatile; graftable on multiple backbones  Biomimetic adhesion mechanism | DOPA→quinone oxidation reduces adhesion over time  Complex multi-step synthesis  Oxidant cytotoxicity; limited scalability | [50] |
| DTG Bioglue (DOPA–Tyrosine Gelatin) | Enzymatic Tyr→DOPA conversion + visible light Ru-based crosslinking | Within 5-10 s of light exposure | Bioinspired enzymatic DOPA generation (no harsh chemicals)  Visible light Ru-crosslinking (safer than UV; deeper penetration)  Gelatin-based: biocompatible, biodegradable, ECM-mimetic, low cost  Rapid crosslinking for immediate structural integrity | Enzymatic conversion requires process optimization  Ru photoinitiator: long-term biocompatibility needs full validation  Unwanted DOPA→quinone oxidation | This work |
